# MAKING CAMPUS GREAT AT ANY AGE: ONE UNIVERSITY’S APPROACH TO AFU PRINCIPLES

**DOI:** 10.1093/geroni/igz038.1252

**Published:** 2019-11-08

**Authors:** Cassandra Barragan, Cassandra Barragan, Andrea Zakrajsek

**Affiliations:** Eastern Michigan University, Ypslianti, Michigan, United States

## Abstract

This presentation will discuss the focused approach to putting AFU principles into practice that we accomplished with our campus-wide steering committee. We performed surveys for students over the age of 40 to learn the perspectives of older learners and what resources they felt were available on campus. By doing this, we were able to understand more about learners pursuing second careers and through a variety of initiatives at the university level, we have improved visibility of older learners and the richness they bring to our campus. We also collected data from our emeritus faculty and staff to learn how they interact with campus in retirement to understand effective ways to actively engage with them as a retired community. Overall, we have been able to effectively use the AFU Initiative to enhance inclusion and diversity to include older learners on campus and make AFU efforts more visible across campus.

